# Deep brain stimulation of medial forebrain bundle modulates noradrenergic activity and feedforward inhibition in rodent model of depression

**DOI:** 10.1038/s41398-025-03577-z

**Published:** 2025-09-29

**Authors:** Zhuo Duan, Yixin Tong, Volker A. Coenen, Máté D. Döbrössy

**Affiliations:** 1 Laboratory of Stereotaxy and Interventional Neurosciences, Department of Stereotactic and Functional Neurosurgery, University Freiburg Medical Center, 79106 Freiburg im Breisgau, Germany; 2 Department of Stereotactic and Functional Neurosurgery, University Freiburg Medical Center, 79106 Freiburg im Breisgau, Germany; 3 Faculty of Medicine, University of Freiburg, 79110 Freiburg im Breisgau, Germany; 4 Faculty of Biology, University of Freiburg, 79104 Freiburg im Breisgau, Germany

**Keywords:** Depression, Molecular neuroscience

## Abstract

Deep Brain Stimulation (DBS) of the superolateral medial forebrain bundle has shown promising long-term anti-depressant effects in treatment-resistant depression patients, although the mechanisms are not clear. The study explored medial forebrain bundle DBS mediated modulation of central noradrenaline transmission in a rodent depression model, the Flinders Sensitive Line (FSL), and in controls, Sprague Dawley (SD) rats. In vivo noradrenergic signaling in the prefrontal cortex (PFC) and nucleus accumbens (NAc) and ultrasonic vocalization were monitored during unilateral mfb-DBS across diverse stimulation parameters. The fiber amount, myelination status, and the activation of ascending projected noradrenergic cell groups (A1, A2, A6) were quantified. Moreover, stimulation induced changes in the parvalbumin-mediated feedforward microcircuitry and neuron activation at PFC and NAc were assessed. FSL rats showed decrease in NA fibers in mfb. Stimulation increased PFC noradrenergic signaling similarly across both groups compared to baseline, but in the NAc, the FSLs had notably higher signaling compared with SDs. FSLs demonstrated more positive affective ultrasonic vocalizations post-DBS than SDs. Brainstem nuclei A1 and A2 had similar noradrenergic neuron density across the experimental groups, and mfb DBS increased neuronal activation in both groups. FSLs had fewer noradrenergic neurons in the A6 nuclei, fewer unmyelinated noradrenergic fibers traversing the mfb, and decreased parvalbumin interneuron activity in both PFC and NAc. DBS normalized parvalbumin interneuron activity in the FSL rats. The study proposes that mfb DBS, via the modulation of the central NA system and the GABAergic inhibitory control of neural excitability, likely contributes to the anti-depressant therapeutic mechanisms reported in both clinical and experimental studies.

## Introduction

Historical and recent experimental data highlights the potential of electrical intracranial self-stimulation of the rodent medial forebrain bundle (“mfb”, when referring to rodent, “MFB” when referring to the human structure) in eliciting reward responses, establishing its role in conditioned learning paradigms [1–3]. Evidence from over the past decade from clinical trials, targeting the superolateral MFB with DBS in Treatment Resistant Depression patients, supports the rapid onset and long-lasting antidepressant effects of continuous and chronic bilateral DBS [4–7], and this is corroborated by preclinical research [8–12]. Despite the clinical and experimental data, the precise neurobiological mechanisms underpinning the therapeutic efficacy of DBS remains unclear. Current theories posit that these effects are driven by the dopaminergic release in the medial prefrontal cortex (mPFC) and nucleus accumbens (NAc) originating from midbrain A10 dopaminergic neurons traversing the mfb, and triggered by mfb DBS evoked antero- and orthodromic activity [3, 13, 14].

In addition to dopamine, noradrenaline (NA) plays a crucial role in depression [15, 16]. Beyond modulating the hypothalamic-pituitary-adrenal axis, increasing evidence underscores its significant role in modulating the cortico-striatum limbic circuit. Central NA provides limbic influence in regulating attention, arousal, pain sensation, memory formation, executive function, motivation, and affective state [17]. Noradrenergic inputs originate from brain stem nuclei, with the A1 (lateral reticular nucleus), A2 (nucleus of the solitary tract), and A6 (locus coeruleus) noradrenergic cell groups contributing to the ascending projection through the mfb [18]. In NAc, NA modulates the parvalbumin (PV) interneuron-mediated feedforward inhibitory microcircuit, which is altered in stressed animal models and plays a pivotal role in abnormal gamma oscillations associated with several psychiatric disorders [19–22].

The mechanisms of action of mfb DBS are multifaceted. Factors such as proximity to the targets, stimulation parameters, and fiber characteristics influence DBS outcomes [23–25]. Studies indicate that myelinated fibers have superior chronaxie and excitability properties, requiring less energy during DBS and responding effectively to microsecond pulse-width stimulation. In contrast, unmyelinated fibers and somatic cells typically require pulse widths in the millisecond range [26]. Consequently, DBS is more likely to initially stimulate myelinated fibers due to their lower activation threshold. Understanding the distribution and myelination profiles of mfb fibers is therefore crucial for comprehending the underlying mechanisms that governs the effects of DBS [14].

In this study, we aimed to better understand the mechanisms of the anti-depressant action of stimulation by specifically examining the effects of mfb DBS on the noradrenergic system in a rodent model of depression (the Flinders Sensitive Line, FSL [10, 27, 28]) and control rats. We investigated the distribution and myelination profiles of ascending noradrenergic projections in the mfb, and present findings on the physiological mapping of NA release in the PFC and NAc under various mfb DBS stimulation conditions and parameters. Furthermore, we describe the activation of NA neurons in the brain stem, as well as the feedforward inhibition circuitry in the PFC and NAc observed in the experimental animals.

## Materials and methods

### Animal

Flinders Sensitive Line (FSL) rats served as the depressive animal model, and age/sex matched Sprague Dawley (SD) rats served as healthy controls. Thirty-nine rats (19 SDs; 20 FSLs) were used in the study. For the fiber distribution and myelination experiment, 6 SDs (3 females, 3 males) and 6 FSLs (3 females, 3 males) were employed. For fiber photometry recording monitoring and subsequent immunohistochemistry, 13 SDs (males) and 14 FSLs (males) were used, with 5 rats in each stimulated group except FSL group for NAc contained 6 rats and 3 in each unstimulated sham group. The animals were aged between 10–12 weeks, weighed between 400–600 grams, and received one-week acclimatization period upon arrival at the facility. All animals were group-housed (3–4 rats/ cage) at the Neurozentrum Freiburg animal facility with *ad libitum* access to food and water, and 12-hour light/dark cycle. The studies were approved by the Regierungspräsidium Freiburg (TVA G21-144 and G20-97) and adhered to the Animals (Scientific Procedures) Act 1986, International Association for Study of Pain [29] and ARRIVE guidelines [30]. Prior to the start of the study, the sample size calculations were confirmed by Institute of Medical Biometry and Statistics (University Freiburg Medical Center, Faculty of Medicine).

### Fiber distribution and myelination

Brain samples were cryosectioned into 40μm thick coronal slices and preserved in an anti-freeze solution at −20 °C. Sections spaced 240μm apart were chosen for immunofluorescent staining. To probe neuronal activation, sections were washed three times in 0.3% PBS (10 min per wash) and then immersed in a blocking buffer containing 5% bovine serum albumin and 0.3% Triton X-100 in 0.3% PBS for an hour. The noradrenergic fibers in mfb were stained with mouse anti-dopamine-beta-hydroxylase antibody (1:700, Chemicon, MAB308) for fiber distribution or double-stained with mouse anti-dopamine-beta-hydroxylase (1:700, Chemicon, MAB308) and rabbit anti-myelin antibodies (1:100, Abcam, ab216590) for fiber myelination.

For myelination staining, an additional antigen retrieval step with 0.5% NaBH4 and 50 °C citrate buffer was performed on the sections. After three washes in 0.3% PBS, sections were exposed to secondary antibodies for 5 h: anti-rabbit 568 (1:200 for distribution and 1:500 for myelin, Life technology, A11011); anti-mouse 488 (1:500, Life technology, A11001) was used only when staining for myelination. Following another three washes in 0.3% PBS, sections were mounted and left to dry in the dark.

Imaging was performed using Axioscan 7 (Zeiss, Germany) paired with ZEN 2.5 software. Pinhole size was set to 30 µm, pixel size to 90 nm, and Z-slice interval to 0.12 µm. The axon myelination images were captured using a Nikon Ti Eclipse microscope equipped with C2 point-scanning confocal unit, 100x objective lens (NA 1.45), appropriate lasers (408, 488 and 561 nm) and emission filters (460/50 nm, 515/30 nm, 600/50 nm). Image quantification was achieved using Image J (NIH, USA). The assessor was blinded during quantification. The mfb anteroposterior (AP) coordinate was delineated based on “The Rat Brain in Stereotaxic Coordinates – 7th edition“ [31], and the boundaries determined according to Nieuwenhuys and collegues [32]. Figure from the aforementioned paper were adapted into a 750 × 950 pixels’ atlas, and the captured images were aligned accordingly. Positive staining was identified using the ImageJ plugin, AxonTracer [33], and translated into yellow pixels. The yellow pixels were recognized, downsized and converted to non-zero elements on the atlas that then were quantified with MATLAB.

### Forced swim test (FST)

At the start of the photometry recording experiment, the depressive-like phenotype was confirmed by FST. The day prior to testing, rats were habituated in a transparent cylinder (60 cm high x 20 cm wide) with water (50 cm deep water, 22 ± 1 °C) for 15 min. The following day, rats were placed in the cylinder for 7 min: “immobility” time was scored in 30-second intervals using a side-positioned video camera and the total immobility time was subsequently quantified. Immobility was defined as: i) no movement of the three out four paws, ii) no struggling, iii) floating behavior. The cylinder was cleaned between subjects to ensure consistent conditions.

### Stereotactic surgery

Anesthesia was induced with 4% isoflurane (with O_2_ as the carrier gas), and rats were placed into a stereotactic apparatus (Stoelting, USA). During the intervention, anesthesia was maintained between 1.5% and 2.0%. AAV9-hsyn-NE2m (YL003008; WZ Biosciences Inc., USA) was injected either into the left mPFC (AP: 3.0 mm, ML: 0.4 mm, DV: −3.2 mm/−3.4 mm) or into the left NAc shell (AP: 1.6 mm/1.2 mm, ML: 1.1 mm, DV: −6.8 mm/−6.9 mm) at two depths using a 2 μl Hamilton syringe. At a rate of 100 nl/min, a total of 1000 nl was injected as 2 x 500 nl deposits (WPI micropump; virus titer 1.77 × 10^13^). Two weeks post-viral injection, DBS electrodes were implanted in the left mfb (AP: −2.8 mm, ML: 1.7 mm, DV: −7.9 mm), and the optic fiber (Doric Lenses, Canada) 200μm above the coordinate where the virus was injected (either in the mPFC or NAc). Dental (Haraeus, Germany) and bone cement (DLine, Germany) were used to secure the implants. Post-operative care included pain management using buprenorphine (0.05 mg/kg) and monitoring for any signs of infection or distress.

### Deep brain stimulation paradigm

Six distinct stimulation parameter combinations were explored, including two frequencies (30 and 130 Hz) and three pulse widths (100, 200, 300μs), applied in a random order on different days. As described elsewhere, the day before the stimulation started, the appropriate current intensity for the individual rats were identified by titration and monitoring of SEEKING behavior during stimulation [9]. The intensity was in the range of 100 - 350μA, which can be explained by the small variations in the electrode placement with respect to the mfb. During the experimental sessions, a 5 s mfb DBS was applied to observe the transmitter release, followed by the observation of the NA spike. Subsequently, NA release over a 50 s span was measured and averaged across 20 repeated trials.

### Fiber photometry recording

Recordings were initiated 4 weeks after viral injection. The virus delivered a gene transcript encoding the alpha-2 adrenergic receptor, which fluoresces in the presence of NA. Prior to each session, all equipment underwent calibration. Rats were connected to the fiber photometry patch cord and a stimulator and placed in a 36 ×36 x 41 cm transparent, open-top box. The recording apparatus comprised of a fluorescence minicube, LEDs, LED drivers, and a fluorescence detector (Doric Lenses® Inc., Canada). Data capture was streamlined using the Synapse Suite Version 94 software in tandem with the RZ5 BioAmp (Tucker-Davis Technologies, USA). Photometric signals were optimized around two pivotal excitation wavelengths: 465 nm tailored for green fluorescence protein (GFP) sensitive excitation and 405 nm designed for isosbestic (GFP-non-sensitive) excitation. Custom-fabricated stimulators produced transistor–transistor logic DBS pulses which were synchronized with the fluorescent signals via the RZ5 BioAmp processor.

Recording lasted 20 mins, and analysis was conducted using pMAT v1.3.24 [34]. The baseline GFP signal, derived from the GRABNA2m sensor activity, was juxtaposed against the isosbestic signal. This comparison facilitated the extraction of the NA fluorescence signal (ΔF/F). DBS events were co-registered and normalized by evaluating a 5 s baseline sampling window that preceded each DBS event. A consistent 100-bin methodology was adopted to deduce key parameters, notably the area under curve and the corresponding time intervals.

### Ultrasonic vocalization

Ultrasonic vocalization patterns reflect the rodent’s affective state, with vocalizations events around 50 kHz associated with positive affective states [35, 36]. Multiple microphones (Dodotronic, UM192K) from different directions were used in recording USV. The monitoring was done simultaneously and under the same conditions as the fiber photometry measurements. Recordings were sampled in the 0–96 kHz range, and the vocalization patterns in the frequency band 40–60 kHz were detected by DeepSqueak [37]. The total number and length of the calls in this band were identified for statistical analysis. Rats categorized as “low callers”, emitting less then five calls during the stimulation/ recording sessions, were excluded from the correlation analysis with NA signaling.

### Immunohistochemistry

The day following the final recording, rats were stimulated with clinical established parameters (130 Hz,100 μs pulse width) and immediately perfused to assess short-term neuronal activation via c-Fos gene expression [38]. The protocol was identical to the one described earlier for the fiber distribution assessment. The primary antibodies used were: rabbit anti-c-Fos (1:200, Cell signaling, #2500); mouse anti-dopamine-beta-hydroxylase (1:700, Chemicon, MAB308); mouse anti-PV (1:1000, Swant, PV235). The secondary antibodies used were: anti-rabbit 488 (1:200, Life technology, A11034); anti-mouse 568 (1:700, Invitrogen, A11004); anti-mouse 405 (1:200, Invitrogen, A31553).

### Statistics analysis

Data analysis were performed using SPSS Statistics or GraphPad Prism. Data were initially screened for normality using the Shapiro-Wilk test. Depending on the distribution of the data, appropriate statistical tests were employed. For normally distributed data, parametric tests such as the student t-test, two-way ANOVA, or Pearson test were used. For non-normal data, non-parametric tests like the Mann-Whitney test, Wilcoxon rank sum test, or Spearman test were applied. When multiple comparisons were made, post-hoc tests with Bonferroni corrections were employed. Data representation was achieved using GraphPad Prism 9.0.0® (GraphPad Software, USA) and MATLAB R2019b® (The MathWorks, USA). Statistical significance was determined at p < 0.05. The statistical analysis concerning the data in the figures are shown in the Supplementary Data section.

## Results

### The distribution and myelination profile of noradrenergic fibers in a rodent model of depression

**Fig. 1:**
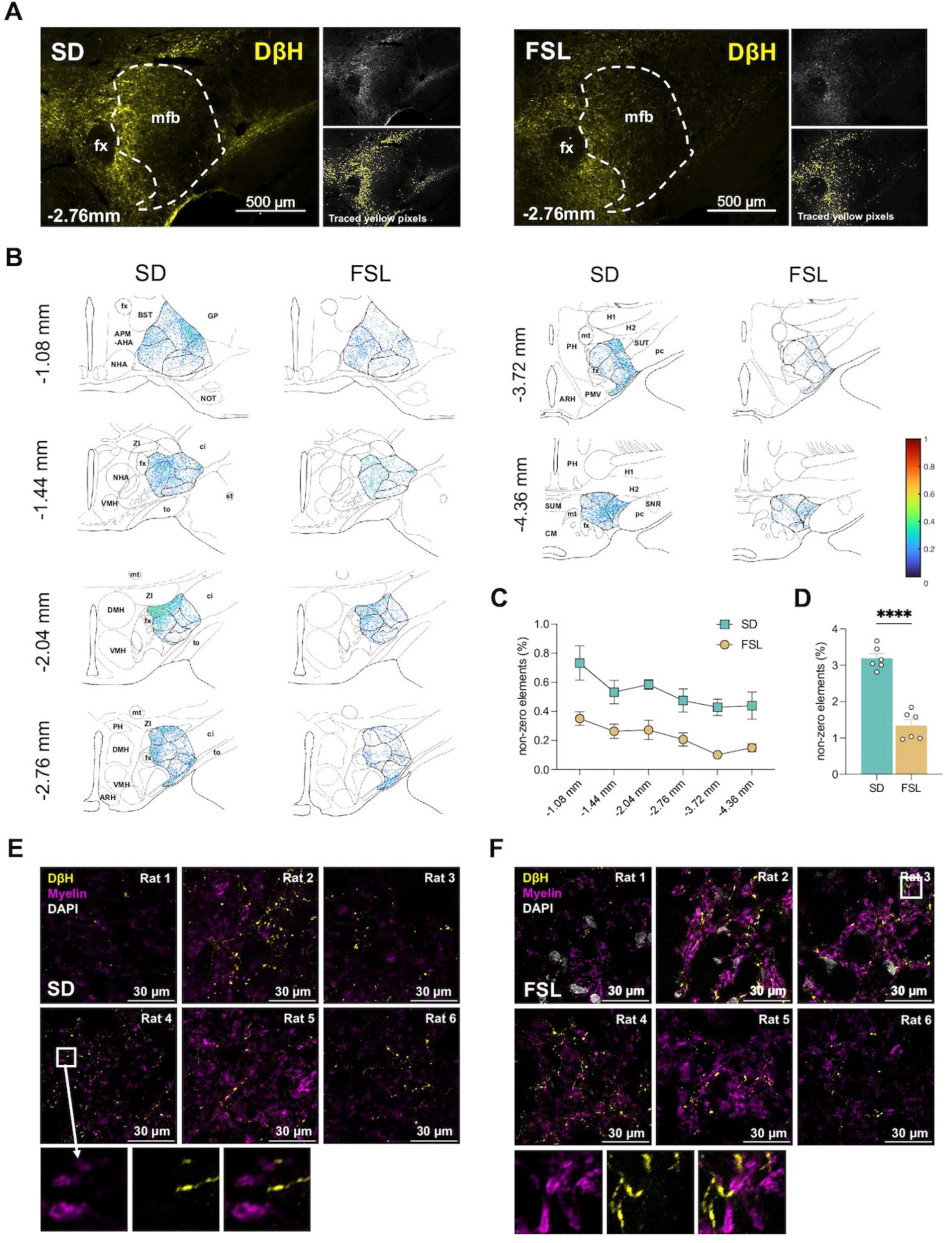
Distribution and myelination of noradrenergic fibers in medial forebrain bundle. **A** Noradrenalin (NA) fibers – identified using Dopamine-ß-Hydraxylase (DßH) as the marker - distribution at AP −2.76 mm (stained by AF488, show in yellow) and traced yellow pixels from a representative Sprague Dawley (SD) and Flinders sensitive line (FSL) rats. **B** Heat map for the distribution of NA fibers in medial forebrain bundle at different AP levels from SD rats (n = 6) and FSL rats (n = 6). **C** The percentage of nonzero yellow pixel in 750 ×950 pixels atlas at each AP levels. **D** The difference of amount of NA fibers between SD and FSL rats, Student’s t-test, *t* = 9.067, df = 10, *P* < 0.0001. **E** Myelination of NA fibers in medial forebrain bundle in SD rats at −2.76 mm AP. **F** Myelination of NA fibers in medial forebrain bundle in FSL rats at −2.76 mm AP. *P < 0.05, **P < 0.01, ***P < 0.001, ****P < 0.0001. Error bars: SEM. Abbreviations: APM-AHA = Area preoptica medialis - Area hypothalamica anterior, ARH = Nucleus arcuatus hypothalami and eminentia mediana, BST = Nucleus interstitialis of the stria terminalis, ci = capsula interna, CM = Corpus mamillare, DMH = Nucleus dorsomedialis hypothalami, fx = fornix, GP = globus pallidum, H1, 2 = Fields of Forel, mt = Fasciculus mamillo-thalamicus, NHA = Nucleus hypothalamicus anterior, NOT = Nucleus tractus olfactorius lateralis, pc = Pedunculus cerebri, PH = Nucleus posterior hypothalami, PMV = premammillary nucleus, ventral, SNR = Substantia nigra, pars reticulata, st = stria terminalis, SUM = Area supramamillaris, SUT = Nucleus subthalamicus, to = tractus opticus, VMH = Nucleus ventromedialis hypothalami, ZI = Zona incerta.

Six sections from Nieuwenhuys’ atlas [32], corresponding to AP levels −1.08 mm, −1.44 mm, −2.04 mm, −2.76 mm, −3.72 mm, and −4.36 mm from Bregma, were analyzed. The noradrenergic fibers projected traversed the posterior and lateral quadrate of the mfb towards the more anterior and superior quadrate of the mfb (Fig. 1A). Specifically, at the −4.36 mm and −3.72 mm level, the fibers were distinctly localized in the lateral quadrant of the mfb. Further anterior at AP −2.76 mm, the level of common implantation site for rodent mfb stimulation, fibers were primarily located in the medial quadrant of the mfb. At −2.04 mm and −1.44 mm level, the NA fibers were localized in the ventromedial mfb segment. Further anterior, at section −1.08 mm, these fibers were predominantly located within both the lateral and medial quadrants of the mfb (Fig. 1B). Overall the six plains of analysis, fewer noradrenergic fibers were observed in the FSLs compared with controls (Fig. 1C, D, *p* < 0.0001).

At the standard implantation site of DBS electrode (AP −2.76), noradrenergic fibers were primarily localized near the fornix (Fig. 1A). Employing fornix as the landmark under a 100x confocal microscope, we observed that the thin, unmyelinated noradrenergic fibers were coursing adjacent to large myelinated axons (Fig. 1E, F). These features suggests that the majority of these slender monoamine fibers are likely to be indirectly stimulated by our DBS protocol at hundred microsecond pulse width range.

### Key insights into strain-specific responses of NA signaling and USV to DBS

**Fig. 2:**
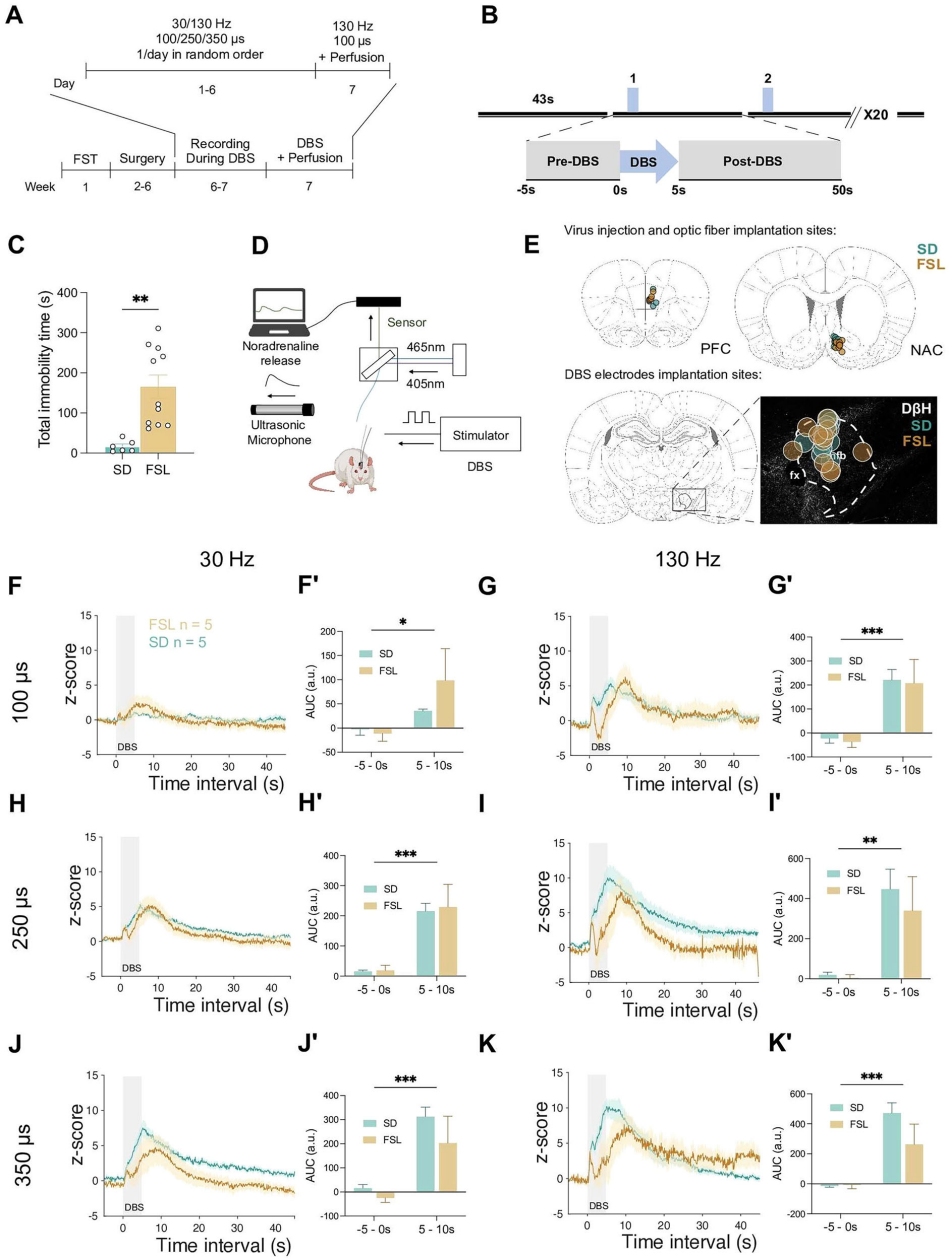
Deep brain stimulation of medial forebrain bundle evoke noradrenalin release in prefrontal cortex (PFC). **A** Timeline of experiment protocol. The rats were first evaluated depressive-like phenotype via the force swim test (FST). During the recording period, six different randomized daily stimulation parameters were used on Days1-6. On the seventh day, the rats were stimulated with 130 Hz 100 us DBS and immediately sacrificed for immunohistochemical examination. DBS, deep brain stimulation. **B** DBS paradigm: 5 s stimulation was given to examine the acute release of NA and ultrasonic vocalization. The stimulation was repeated 20 times with 50 s interval. **C** Total immobility time in force swim test between Sprague Dawley (SD, n = 6) and Flinders Sensitive Line (FSL, n = 11) rats; Student’s t-test, *t* = 3.685, df = 15, *P* = 0.0022. **D** Schematic drawing of experiment set up. **E** Schematic drawing of virus injection/ optic fiber implantation sites in the mPFC (left) and NAC (right) in SD and FSL animals; the DBS electrode implantation (below) in the medial forebrain bundle (mfb) is also shown, and the distribution of the sites is illustrated in more detail (magnification). Dopamine-ß-Hydroxylase (DßH) staining identified the noradrenergic component in the mfb. (**F-K**) NA release 5 s prior to DBS, 5 s during DBS, and 50 s post-DBS under different conditions and across the experimental groups; **(F´-K´)** Pre-DBS vs DBS release under different conditions and across the experimental groups. (**F, F´)** NA release in PFC at 30 Hz 100 μs. (**G, G´**)130 Hz 100 μs. (**H, H´**) 30 Hz 250 μs. (**I, I´**) 130 Hz 250 μs. (**J, J´**) 30 Hz 350 μs. (**K, K´**) 130 Hz 350 μs; animal strains versus stimulation condition (pre DBS, −5 - 0 and during DBS, 0 - 5). *P < 0.05, **P < 0.01, ***P < 0.001, ****P < 0.0001. Error bars: SEM.

**Fig. 3:**
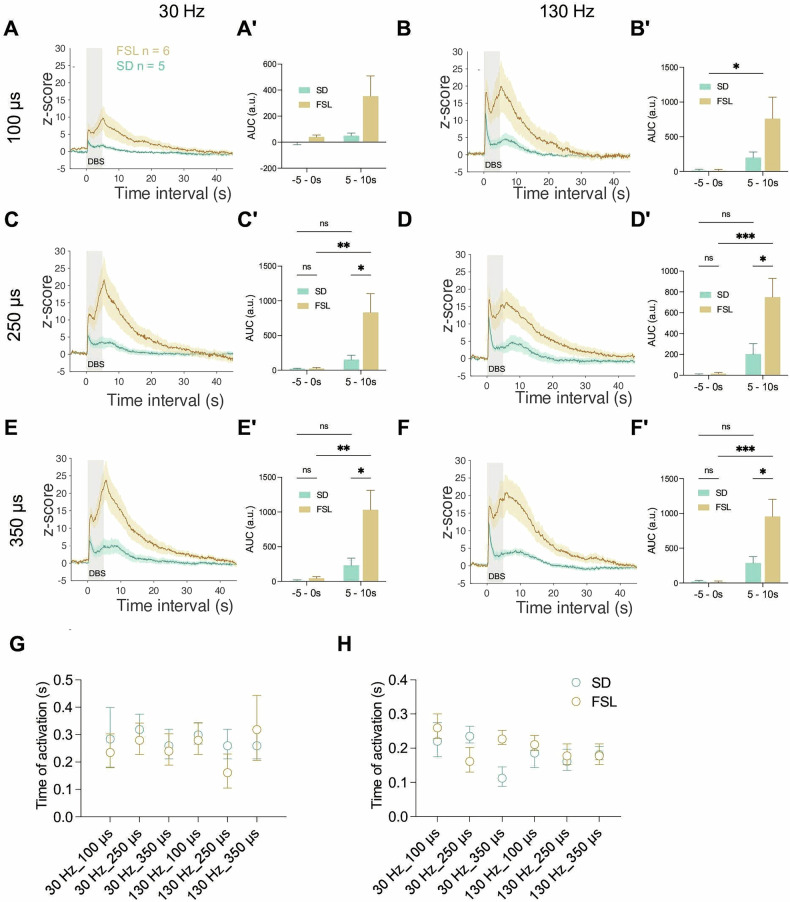
Deep brain stimulation of medial forebrain bundle evoke noradrenalin release in nucleus accumbens (NAc). **A-F** NA release 5 s prior to DBS, 5 s during DBS, and 45 s post-DBS under different conditions and across the experimental groups; **(A´-F´)** Pre-DBS vs DBS release under different conditions and across the experimental groups. (**A, A´**) NA release in NAc at 30 Hz 100 μs. (**B, B´**)130 Hz 100 μs. (**C, C´**) 30 Hz 250 μs. (**D, D´**) 130 Hz 250 μs. (**E, E´**) 30 Hz 350 μs. (**F, F´**) 130 Hz 350 μs; animal strains versus stimulation condition (pre DBS, −5 - 0 and during DBS, 0 - 5). **G** The initial time of signal activation under diverse stimulation parameters at PFC in SD and FSL rats. **H** The initial time of signal activation under diverse stimulation parameters at NAc in SD and FSL rats. *P < 0.05, **P < 0.01, ***P < 0.001, ****P < 0.0001, ns, non-significant. Error bars: SEM.

The experimental timelines for photometry recording and DBS stimulation paradigm are depicted in Fig. 2A and B, respectively. Before surgical intervention, both FSL and SD rats underwent phenotyping by the FST. FSL demonstrated significantly higher total immobility times compared to their SD counterparts (Fig. 2C, p = 0.002). The assessments were performed by integrating the photometry system and ultrasonic vocalization as illustrated in Fig. 2D. The optic fiber was placed unilaterally in the left PFC or NAc at the viral injection site and DBS electrode was implanted at ipsilateral mfb (Fig. 2E). Baseline monitoring started 5 s prior to the stimulation (-5-0s). The stimulation (0–5 s) prompted an immediate release and binding (signaling) of NA in both the PFC and NAc. Upon initiation of stimulation, we observed an early rapid peak in transmitter signaling, which then quickly decreased within 1 – 2 s. The peak, or peaks, in signalling were typically observed during the first 10 s, and mainly in the 5 s post DBS (5–10 s). Following the peak release, the signaling gradually returned to baseline over the next 40 s. The release patterns observed were dependent on the stimulation parameters used, the region monitored, and the experimental group. The changes in NA signaling over the entire monitoring timeline are shown in Figs. 2F–K and 3A–F, for PFC and NAc, respectively.

In the PFC, either increasing the frequency (from 30 to 130 Hz) or the pulse-width (from 100 to 250 to 350µs), resulted in the significant increase in NA signaling under all stimulation conditions (Fig. 2F´, 30 Hz 100 μs, *p* = 0.024; Fig. 2H´, 30 Hz 250 μs, *p* = 0.001; Fig. 2J´, 30 Hz 350 μs, *p* = 0.001; Fig. 2G´, 130 Hz 100 μs, *p* = 0.003; Fig. 2I´, 130 Hz 250 μs, *p* = 0.003; Fig. 2K´, 130 Hz 350 μs, *p* = 0.002). The stimulation evoked release observed were quantitatively similar across the experimental groups, although the maximum release point in the FSLs seemed to be achieved with a few seconds delay compared to the controls.

In the NAc, switching to a higher frequency or to longer pulse-width selectively and significantly affected FSL NA signaling more than it did the controls. Not only was there more release observed in the FSLs, but systematically with all six stimulation conditions, a second “peak” - associated with the end of the stimulation - also appeared suggestive of a stimulation induced “direct”, followed by an “indirect” release mechanism (Fig. 3A–F). Stimulation, assessed by comparing pre (-5-0s) and post-stimulation (5–10 s) release, exerted a significant influence on NA signaling under all parameters except at 30 Hz 100μs (Fig. 3A´, 30 Hz 100 μs, *p* = 0.056; Fig. 3C´, 30 Hz 250 μs, *p* = 0.01; Fig. 3E´, 30 Hz 350 μs, *p* = 0.003; Fig. 3B´, 130 Hz 100 μs, *p* = 0.03; Fig. 3D´, 130 Hz 250 μs, *p* = 0.002; Fig. 3F´, 130 Hz 350 μs, *p* = 0.002). Moreover, the DBS evoked increase in NA signaling was significantly greater amongst the FSL animals compared to controls, especially at longer pulse-widths (Fig. 3C´, 30 Hz 250 μs, *p* = 0.042; Fig. 3E´, 30 Hz 350 μs, *p* = 0.035; Fig. 3D´, 130 Hz 250 μs, *p* = 0.003; Fig. 3F´, 130 Hz 350 μs, *p* = 0.036).

The initiation/onset time of the NA signaling was analyzed. The SDs and FSLs showed similar time of release in both PFC and NAc (Fig. 3G, H). For PFC, the signaling started at 0.28 ± 0.12 s in SDs and at 0.25 ± 0.16 s in FSLs. For NAc, the signaling started at 0.20 ± 0.08 s in SDs and 0.18 ± 0.08 s in FSLs. Five data points obtained from the PFC of FSL animals exhibited signaling onset at 0.06 s, which corresponds to the minimal temporal resolution of the photometry device. This instantaneous response suggests a direct activation of noradrenergic fibers in mfb.

**Fig. 4:**
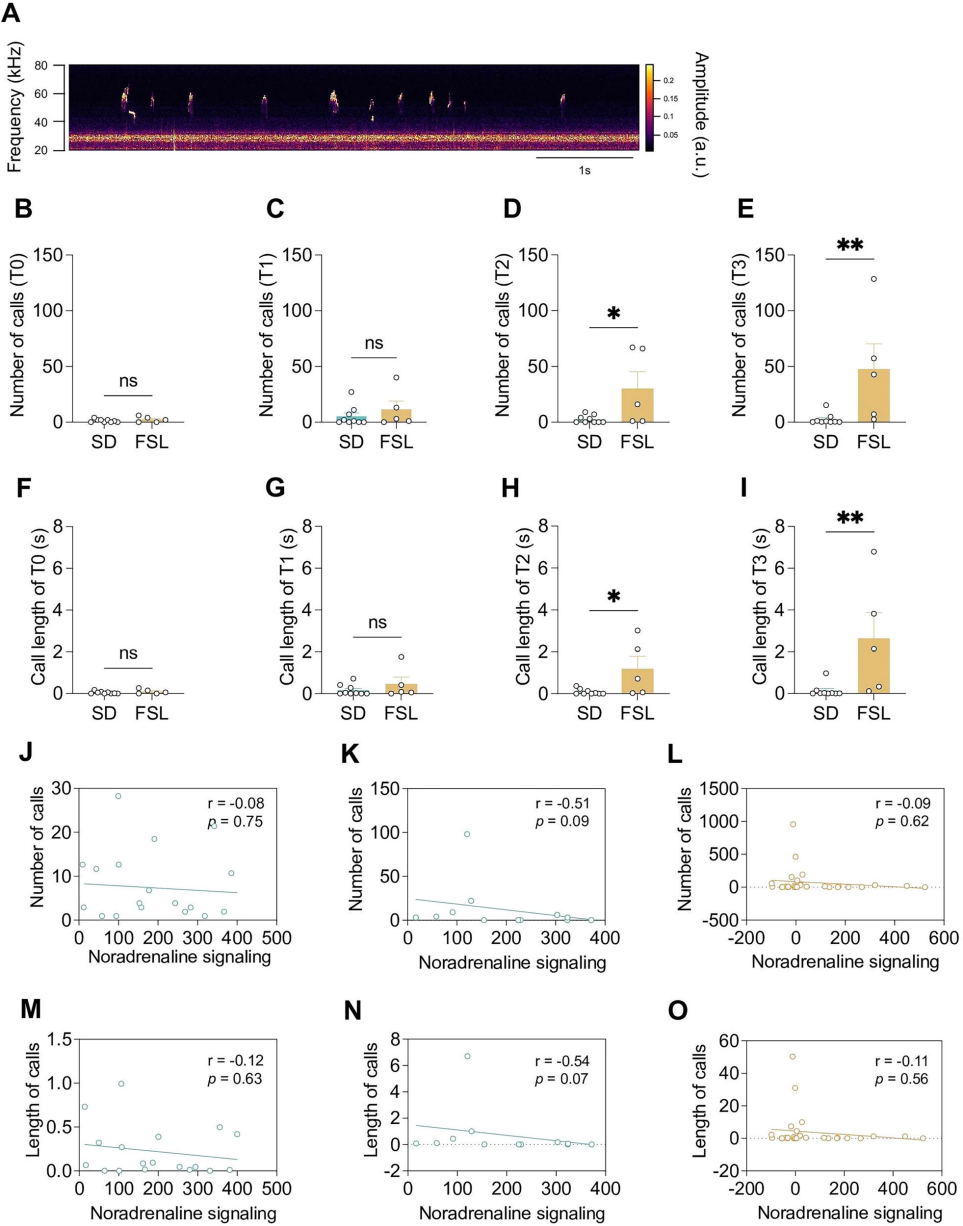
Ultrasonic vocalization of Sprague Dawley and Flinders sensitive line rats. **A** Representative picture of positive affective vocalization (40 kHz to 60 kHz) from a FSL rat. **B** Number of calls for pre- DBS (T0). (**C**) during DBS (T1). (**D**) 5 s after DBS (T2). (**E**) post DBS (T3). (**F**) Length (s) of call for pre-DBS (T0). **G**, during DBS (T1) (**H**) 5 s after DBS (T2). (**I**) post DBS (T3). **J** Correlation between number of calls and NA signalling at PFC of SD. (**K**) NAc of SD. (**L**) PFC of FSL. (**M**) Correlation between length of calls and NA signalling at PFC of SD. **N** NAc of SD. (**O**) PFC of FSL. *P < 0.05, **P < 0.01, ***P < 0.001, ****P < 0.0001, ns, non-significant. Error bars: SEM.

Vocalizations within the 40 kHz to 60 kHz range representing a positive affective state were analyzed (Fig. 4A). The number and length of calls between the FSL and SD rats were compared across four distinct time intervals: T0 (pre-DBS), T1 (during DBS), T2 (5–10 s post-DBS), and T3 (averaged over the final 40 s post-DBS). No significant differences emerged between the SDs and FSLs during the T0 and T1 intervals in either the number of calls (Fig. 4B, C; T0 and T1, respectively) or the length of calls, (Fig. 4F, G; T0 and T1, respectively), Post-DBS, FSLs’ vocalization increased significantly compared to SDs in both the absolute number of the calls (Fig. 4D, T2, *p* = 0.047 ; Fig. 4E, T3, *p* = 0.007) and the length of the calls (Fig.4H, T2, *p* = 0.038; Fig. 4I, T3, *p* = 0.007). The number and length of calls were analyzed for correlation with change in the stimulation evoked NA signaling at PFC of SDs/ FSLs, as well as at NAc of SDs. However, no correlations were identified in either the number nor the length of the calls with the NA signaling at PFC of SD (Fig. 4J, M), NAc of SD (Fig. 4K, N), PFC of FSL (Fig. 4L, O).

### mfb DBS activates A1, A2 noradrenergic cell groups and disinhibits neurons in PFC and NAc

**Fig. 5:**
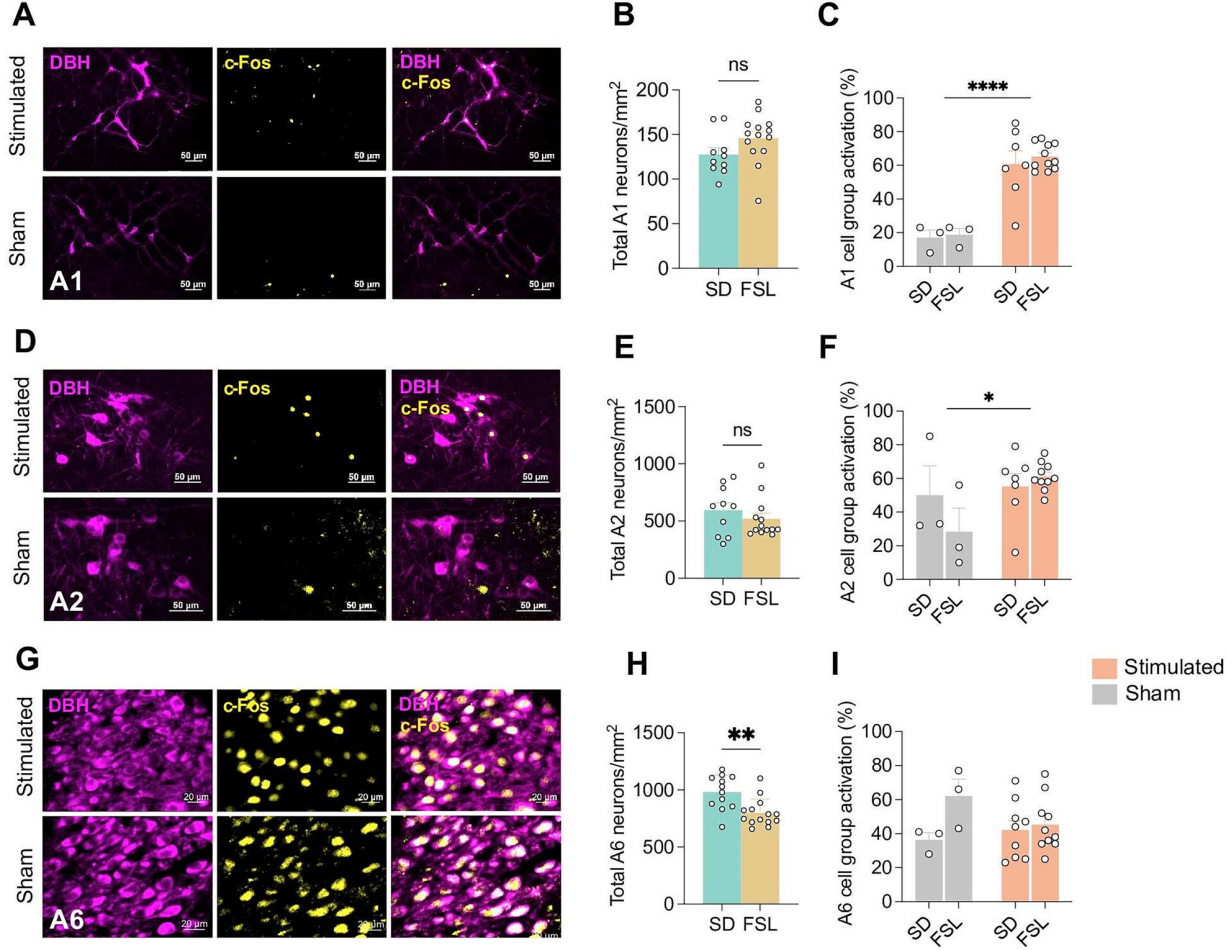
Activation of noradrenergic cell group by unilateral medial forebrain bundle stimulation. **A** Immunohistochemical analysis of A1 noradrenergic (NA) cell groups without (sham) and with medial forebrain bundle stimulation (stimulated) the AF488 showed in yellow, AF568 showed in magenta. **B** Overall density of A1 NA neurons in Sprague Dawley (SD, n = 10) and Flinders Sensitive Line rats (FSL, n = 14). **C** A1 NA neurons activation in SD rats and FSL rats after 20 times 5 s medial forebrain bundle stimulation compared with unstimulated sham animals. **D** Immunohistochemical analysis of A2 NA cell groups from sham and stimulated animals. **E** Overall density of A2 noradrenergic neurons in SD (n = 10) and FSL (n = 13) rats. **F** A2 NA neurons activation in SD rats and FSL rats after 20 times 5 s medial forebrain bundle stimulation compared with unstimulated sham animals. **G** Immunohistochemical analysis of A6 NA cell groups from sham and stimulated. **H** Overall density of A6 noradrenergic neurons in SD (n = 12) and FSL (n = 14) rats. (**I**) A6 NA neurons activation in SD rats and FSL rats after 20 times 5 s medial forebrain bundle stimulation compared with unstimulated sham animals. *P < 0.05, **P < 0.01, ***P < 0.001, ****P < 0.0001, ns, non-significant. Error bars: SEM.

To investigate whether and to what degree mfb DBS activated noradrenergic cell groups in the brainstem, we used a double staining approach staining for c-Fos expression and Dopamine beta-hydroxylase (DßH), a maker of noradrenergic neurons. The noradrenergic cell groups A1, A2, and A6, projecting from the brainstem via the mfb to forebrain structures were stained (Fig. 5A, D, G). While the cell density of A1 and A2 neurons displayed no significant differences between SDs and FSLs (Fig. 5B, E), the A6 neurons in FSLs exhibited a notably lower normalized total cell count compared to SDs (Fig. 5H; p = 0.0022). In terms of the stimulation impact, significant increases in the activity were observed equally in both experimental groups from baseline activity to the post-stimulation activity in both the A1 (Fig. 5C; sham, M_SD_ = 17.0 ± 4.6%, M_FSL_ = 18.7 ± 3.8%; stimulated, M_SD_ = 60.7 ± 7.9%, M_FSL_ = 65.1 ± 2.3%; *p* < 0.0001) and the A2 noradrenergic hubs (Fig. 5F; sham, M_SD_ = 50.0 ± 17.5%, M_FSL_ = 28.3 ± 14.1%; stimulated, M_SD_ = 55.3 ± 7.6%, M_FSL_ = 61.1 ± 2.6%; *p* = 0.037). No significant stimulation induced changes were observed in the activity of A6 noradrenergic neurons in either experimental groups (Fig. 5I; sham, M_SD_ = 36.3 ± 4.2%, M_FSL_ = 62.0 ± 10.0%; stimulated, M_SD_ = 42.1 ± 5.6%, M_FSL_ = 45.3 ± 5.0%; n.s.).

**Fig. 6:**
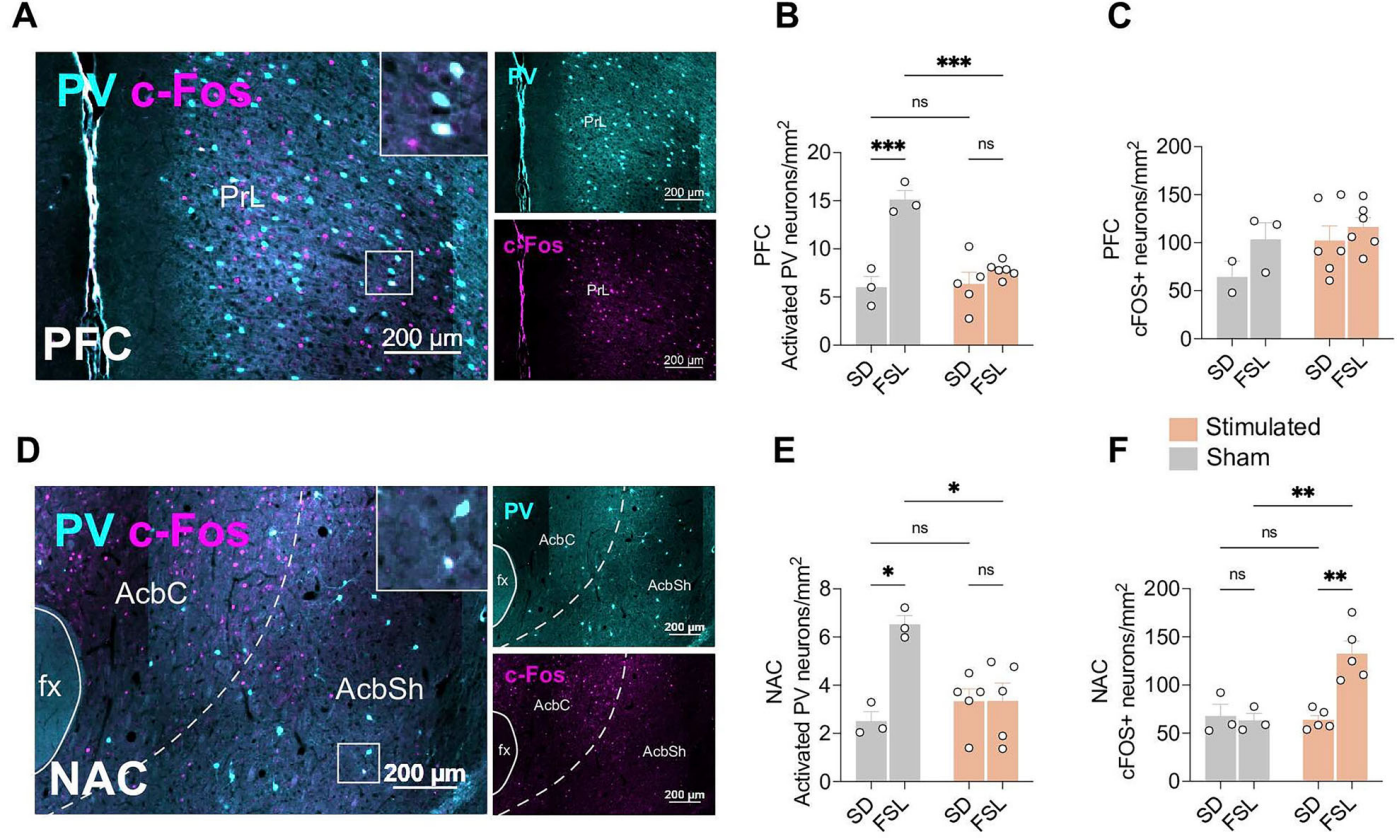
Activation of parvalbumin (PV) interneuron and overall c-Fos expression by unilateral mfb DBS in prefrontal cortex (PFC) and nucleus accumbens (NAc). **A** Activated parvalbumin interneuron in PFC from representative animal, AF568 showed in magenta, AF405 showed in cyan. **B** The density of active PV interneurons with and without DBS at PFC in SD rats and FSL rats, two-way ANOVA. **C** The density of c-Fos positive neurons at PFC in SD rats and FSL rats, two-way ANOVA. **D** Activated parvalbumin interneurons in NAc from representative animal, AF568 showed in magenta, AF405 showed in cyan. **E** The density of active PV interneurons with and without DBS at NAc in SD rats and FSL rats. **F** The density of c-Fos positive neuron at NAc in SD rats and FSL rats. *P* < 0.05, **P < 0.01, ***P < 0.001, ****P < 0.0001, ns, non-significant. Error bars: SEM.

To elucidate the local effect at PV interneurons in the PFC and NAc, baseline activated density and activation in stimulated and sham conditions were compared in SDs and FSLs. The activation was measured by colocalization of PV interneuron with c-Fos staining (Fig. 6A, D). Two-way ANOVAs, analyzing possible interactions between stimulation conditions and experimental group on PV interneuron activation, showed a significant interaction between stimulation and experimental groups in both the PFC (Fig. 6B; *p* = 0.001) and the NAc (Fig. 6E; *p* = 0.03). Post-hoc analysis revealed FSLs has a significant higher baseline activity of PV interneuron as SD in both PFC (*p* < 0.0001) and NAc (*p* = 0.003). The elevated activity in the FSLs was significantly reduced to the baseline after the stimulation (PFC, *p* = 0.0002; NAc, *p* = 0.008). The stimulation had no impact on the activated PV neuron numbers in the SDs.

The overall c-Fos-positive neurons were also quantified in the areas of interest (Fig. 6C, F). A two-way ANOVA revealed a statistically significant interaction between stimulation conditions and experimental groups in the NAc (Fig. 6F; *p* = 0.005). Post-hoc analysis showed that the overall c-Fos expression in FSL animals significantly increased following stimulation (*p* = 0.001), whereas no significant change was observed in SD animals.

Additionally, we quantified the contralateral (right) side and observed that, despite unilateral stimulation, both SDs and FSLs exhibited similar effects on both sides of the brain. Specifically, the activation of NA neurons and the local effects in the PFC and NAc (including PV interneurons and overall neuronal activity) were comparable across the examined brain nuclei on both hemispheres.

## Discussion

The study examined mfb DBS evoked NA signaling in the NAc and mPFC in a rodent depression model, the Flinders Sensitive Line (FSL), and in controls, Sprague Dawley (SD) rats. The results confirmed that mfb DBS can modulate the activation of noradrenergic nuclei, and influence NA signaling in PFC and NAc. The NA signaling increased as a function of increasing stimulation frequencies and pulse widths. The study reports experimental group differences in myelination patterns, in stimulation mediated activation of accumbal PV interneuron activity and in mfb DBS induced NA signaling in the NAc and PFC. The anti-depressant therapeutic mechanisms of mfb DBS observed clinically and in experimental models likely has a NA component. Furthermore, the data suggests that a differential functioning of downstream regulatory mechanisms – e.g. PV interneuron mediated feedforward inhibition – could explain the differences in the behavior patterns observed across experimental groups.

The FSL is a validated rodent depression model with both pathophysiological and spontaneously emerging “depressive-like” phenotype [27, 28, 39]. We have shown using multiple neurotransmitter monitoring techniques that mfb DBS induces acute and enduring secretion of dopamine in forebrain structures such as the mPFC and NAc, and the release patterns are different in the FSL and control animals [23, 25, 40]. The current study demonstrated this also to be the case for NA transmission, a neurotransmitter implicated in the neuropathophysiology of depression associated with clinical symptoms such as decreased alertness, low energy, inattention, reduced concentration and cognitive ability [41]. Although the temporal resolution of fiber photometry is limited, the data related to the mfb DBS evoked initiation of NA signaling suggests this occurred via both direct, but primarily via the indirect activation of the dominantly unmyelinated NA projections. The thin unmyelinated monoamine fibers require a high current density to be activated, and the anti-depressant effect is likely to be the sum of both the direct and indirect activation of the monoamine axons and other neurotransmitter and neuromodulator systems traversing the mfb [14].

Although the temporal resolution of photometry is limited, the initiation timing revealed that NA signaling was primarily induced via indirect activation of NA neurons by mfb stimulation due to the extended response time to the stimulation. In few stimulation trials, the signaling at PFC of FSL started before 60 ms as the minimal measure of photometry. Direct activation of nearby NA fibers may occur due to their distinctive varicosities, which are larger than intervening axon segments [42]. This was histologically observed through confocal imaging. The varicosities’ size are comparable to some of the myelinated neurons within the mfb. Large myelinated neurons in the mfb are more readily activated, potentially driving brainstem noradrenergic cells and leading to indirect activation.

Converging data from our group suggest deregulation of fast-spiking GABAergic interneurons in mPFC and NAc as a key mechanism in the pathophysiology of the FSL depression model, and that stimulation modulates Gamma oscillations through GABAergic mechanisms. Previous acute electrophysiological recordings from anesthetized animals indicate that mfb DBS enhances high Beta (21–30 Hz) and low Gamma (30–48 Hz) oscillations in the mPFC and NAc of FSL rats - compared to controls - with distinct baseline spectral power differences reflecting trait-like neurophysiological biomarkers of the depression model [24]. Stimulation-mediated increase in gene expression of biomarkers associated with GABAergic transmission (e.g. *GABAA, GAD1*) have also been shown [43]. Manz and colleagues demonstrated that, in the shell of NAc, NA modulated the local fast-spiking GABAergic PV interneuron mediated feedforward microcircuitry via the presynaptic alpha 2 adrenergic receptor [44]. In our study, we recorded the in vivo signaling at alpha-2 adrenergic receptor in PFC and NAc, and report decreased PV interneuron activity in both structures. The reduced PV activity was normalized in the depressive model after our 20 min burst stimulation. Moreover, the overall neuron activation in NAc measured by c-Fos colocalization also increased in FSLs. This is possibly due to the local disinhibition effect mediated by the PV interneurons on NA signaling in the NAc. This modulation is crucial as it underscores the broader implications of NA in regulating neural circuits, especially in the context of depressive disorders. Interestingly, the effect of unilateral DBS appeared to have bilateral effects in both NA cell groups and PV interneuron activation. It indicates the presence of collateral innervations that are also modulated by mfb DBS to produce bilateral effects as reported elsewhere [43].

Noradrenergic projections originating from brainstem areas A1, A2, and A6 pass through the mfb and contribute to central noradrenergic functions. Quantification of noradrenergic neurons in FSL and SD animals showed similar neuronal densities in their A1 and A2 areas. However, significantly lower noradrenergic neuronal density was identified in A6 of the FSL animals compared to SDs. The lower neuronal density in A6, could explain the reduced number of noradrenergic fibers observed specifically in the mfb of the FSL animals. Area A6, the locus coeruleus, is the main source of NA in the brain and is related to a variety of functions such as arousal, anxiety, stress and fear [45]. In animal models, experimentally induced reduction in A6/LC neurons has shown to cause depression-like behaviors [46], and the phenotype previously reported associated with the FSL [27, 28] could in part be related to differences in the noradrenergic system documented in the current study. Medial forebrain bundle DBS is a non-selective form of neurostimulation which can modulate the activity of fiber projections traversing the bundle both ortho- and antidromically. Similarly low baseline activities amongst the NA neurons were detected in the A1, A2, and A6 nuclei in the FSL and SD animals, and mfb DBS equally increased the proportion of active neurons in A1 and A2 across the groups. No apparent differences in the proportion of activated A6 noradrenergic neurons were observed.

The current study has numerous limitations. Although the study focused on and provided evidence of mfb stimulation-induced NA signaling, it did not investigate the impact of prolonged stimulation on signaling dynamics. Longer stimulation durations are necessary to evaluate the continuous release of NA and its relationship with behavior. We observed a bilateral phenomenon in NA cell group activation and PV modulation. To further support these findings, bilateral recordings of NA signaling are required. Additionally, USV responses varied significantly among individual subjects, making it challenging to identify a linear relationship. Conducting larger-scale experiments with comparable baseline rodents could improve the accuracy and reliability of the results. While we successfully demonstrated in vivo signaling, fiber photometry measures only the relative changes in NA levels, and the absolute amount cannot be quantified. Techniques, such as calcium imaging and dual-channel simultaneous recordings, could provide more precise measurements of cell group compared to c-Fos analysis. Finally, the structure, cellular makeup/ distribution/ density and functional connectivity of the rodent mfb differs from the human MFB [47, 48]. As such, these findings should be interpreted with caution when drawing parallels to clinical scenarios.

In conclusion, in the context of a rodent model of depression, our findings provide novel understanding of the noradrenergic system’s response to mfb DBS. The data indicates that stimulation of this key fiber bundle linking midbrain and forebrain structures can evoke central NA release. Furthermore, the study indicates differential response to the DBS across the experimental depression model and the control groups likely due to divergence in brainstem noradrenergic nuclei and in the NA mediated regulation of inhibitory micro-circuitries in the NAc. Better targeting of therapeutic interventions can be achieved via future studies delving deeper into mechanisms underlying stimulation-mediated monoaminergic signaling, and an improved understanding of GABAergic inhibitory control of neural excitability in healthy controls and experimental models of depression.

## Supplementary information

Supplementary data

## Data Availability

We confirm that all data supporting the findings of this study are available within the paper, or submitted in the Supplementary data file. Raw data collected during the study by the authors are available from the corresponding author on request.
