# Supplementary material for: Deep brain stimulation of medial forebrain bundle modulates noradrenergic activity and feedforward inhibition in rodent model of depression
Source: Transl Psychiatry. 2025 Sep 29;15:343. doi: 10.1038/s41398-025-03577-z (PMC12480665; doi:10.1038/s41398-025-03577-z)
Supplement: Supplementary file 1 — Supplementary data [file 41398_2025_3577_MOESM1_ESM.docx]

**Supplementary**

**Deep brain stimulation of medial forebrain bundle modulates noradrenergic activity and feedforward inhibition in rodent model of depression**

Zhuo Duan^1,2,4^, Yixin Tong^1,2,5,6^, Volker A. Coenen^1,2,4^ and **Máté**D. Döbrössy^1,2,3^

**Author affiliations:**

^1^Laboratory of Stereotaxy and Interventional Neurosciences, Department of Stereotactic and Functional Neurosurgery, University Freiburg Medical Center, 79106 Freiburg im Breisgau, Germany

^2^Department of Stereotactic and Functional Neurosurgery, University Freiburg Medical Center, 79106 Freiburg im Breisgau, Germany

^3^Faculty of Biology, University of Freiburg, 79104 Freiburg im Breisgau, Germany

^4^Faculty of Medicine, University of Freiburg, 79110 Freiburg im Breisgau, Germany

^5^Department of Ophthalmology, University Hospital of Bern, University of Bern, 3010 Bern, Switzerland

^6^Department for BioMedical Research, University of Bern, 3008 Bern, Switzerland

| **Table S1 Statistical analysis** | | | | | | | | | | |
| --- | --- | --- | --- | --- | --- | --- | --- | --- | --- | --- |
| **Figure** | **Subject** | **Group** | | **Mean** | **SEM** | **n** | **Method** | **Value** | | **p** |
| Fig. 1D | Non-zero elements | SD |  | 3.19 | 0.13 | 6 | t-test |  | t=9.067, df=10 | <0.0001 |
|  |  | FSL |  | 1.34 | 0.16 | 6 |  |  |  |  |
| Fig. 2C | Total immobility time | SD |  | 16.90 | 5.93 | 6 | t-test |  | t=3.685, df=15 | 0.0022 |
|  |  | FSL |  | 165.53 | 29.18 | 11 |  |  |  |  |
| Fig. 2F | AUC | SD | -5 – 0s | -2.78 | 12.04 | 5 | two-way ANOVA | time x animal strain | F (1, 8) = 1.823 | 0.2140 |
|  |  |  | 5 – 10s | 35.55 | 3.72 |  |  | time | F (1, 8) = 7.793 | 0.0235 |
|  |  | FSL | -5 – 0s | -11.25 | 15.86 | 5 |  | animal strain | F (1, 8) = 0.4594 | 0.5170 |
|  |  |  | 5 – 10s | 98.86 | 65.39 |  |  |  |  |  |
| Fig. 2H | AUC | SD | -5 – 0s | 15.71 | 4.58 | 5 | two-way ANOVA | time x animal strain | F (1, 8) = 0.01527 | 0.9047 |
|  |  |  | 5 – 10s | 215.37 | 26.10 |  |  | time | F (1, 8) = 23.56 | 0.0013 |
|  |  | FSL | -5 – 0s | 19.15 | 16.79 | 5 |  | animal strain | F (1, 8) = 0.04882 | 0.8307 |
|  |  |  | 5 – 10s | 229.25 | 75.18 |  |  |  |  |  |
| Fig. 2J | AUC | SD | -5 – 0s | 15.71 | 14.72 | 5 | two-way ANOVA | time x animal strain | F (1, 8) = 0.4597 | 0.5169 |
|  |  |  | 5 – 10s | 312.33 | 39.61 |  |  | time | F (1, 8) = 26.29 | 0.0009 |
|  |  | FSL | -5 – 0s | -24.66 | 18.54 | 5 |  | animal strain | F (1, 8) = 1.202 | 0.3049 |
|  |  |  | 5 – 10s | 202.68 | 111.61 |  |  |  |  |  |
| Fig. 2G | AUC | SD | -5 – 0s | -22.61 | 19.80 | 5 | two-way ANOVA | time x animal strain | F (1, 8) = 7.458e-005 | 0.9933 |
|  |  |  | 5 – 10s | 221.05 | 43.72 |  |  | time | F (1, 8) = 17.69 | 0.0030 |
|  |  | FSL | -5 – 0s | -36.75 | 23.00 | 5 |  | animal strain | F (1, 8) = 0.06441 | 0.8061 |
|  |  |  | 5 – 10s | 207.91 | 98.41 |  |  |  |  |  |
| Fig. 2I | AUC | SD | -5 – 0s | 19.73 | 13.58 | 5 | two-way ANOVA | time x animal strain | F (1, 8) = 0.2615 | 0.6229 |
|  |  |  | 5 – 10s | 447.08 | 99.82 |  |  | time | F (1, 8) = 17.88 | 0.0029 |
|  |  | FSL | -5 – 0s | 5.28 | 15.25 | 5 |  | animal strain | F (1, 8) = 0.3248 | 0.5844 |
|  |  |  | 5 – 10s | 340.42 | 168.69 |  |  |  |  |  |
| Fig. 2K | AUC | SD | -5 – 0s | -14.81 | 7.63 | 5 | two-way ANOVA | time x animal strain | F (1, 8) = 1.716 | 0.2266 |
|  |  |  | 5 – 10s | 471.38 | 68.31 |  |  | time | F (1, 8) = 21.38 | 0.0017 |
|  |  | FSL | -5 – 0s | -8.47 | 24.31 | 5 |  | animal strain | F (1, 8) = 2.016 | 0.1934 |
|  |  |  | 5 – 10s | 263.09 | 135.00 |  |  |  |  |  |
| Fig. 3A | AUC | SD | -5 – 0s | -2.43 | 16.50 | 5 | two-way ANOVA | time x animal strain | F (1, 9) = 2.436 | 0.1530 |
|  |  |  | 5 – 10s | 50.00 | 20.13 |  |  | time | F (1, 9) = 4.791 | 0.0564 |
|  |  | FSL | -5 – 0s | 40.64 | 14.26 | 6 |  | animal strain | F (1, 9) = 3.671 | 0.0876 |
|  |  |  | 5 – 10s | 353.63 | 155.38 |  |  |  |  |  |
| Fig. 3C | AUC | SD | -5 – 0s | 20.03 | 7.68 | 5 | two-way ANOVA | time x animal strain | F (1, 18) = 4.799 | 0.0419 |
|  |  |  | 5 – 10s | 152.51 | 65.82 |  |  | time | F (1, 18) = 9.321 | 0.0068 |
|  |  | FSL | -5 – 0s | 24.87 | 13.07 | 6 |  | animal strain | F (1, 18) = 4.938 | 0.0393 |
|  |  |  | 5 – 10s | 830.41 | 272.28 |  |  |  |  |  |
| Fig. 3E | AUC | SD | -5 – 0s | 9.49 | 14.25 | 5 | two-way ANOVA | time x animal strain | F (1, 9) = 6.188 | 0.0346 |
|  |  |  | 5 – 10s | 233.61 | 101.83 |  |  | time | F (1, 9) = 15.61 | 0.0033 |
|  |  | FSL | -5 – 0s | 46.69 | 23.11 | 6 |  | animal strain | F (1, 9) = 6.062 | 0.0360 |
|  |  |  | 5 – 10s | 1032.43 | 279.16 |  |  |  |  |  |
| Fig. 3B | AUC | SD | -5 – 0s | 15.42 | 17.77 | 5 | two-way ANOVA | time x animal strain | F (1, 9) = 2.444 | 0.1524 |
|  |  |  | 5 – 10s | 200.51 | 81.61 |  |  | time | F (1, 9) = 6.704 | 0.0293 |
|  |  | FSL | -5 – 0s | 12.49 | 17.45 | 6 |  | animal strain | F (1, 9) = 2.698 | 0.1349 |
|  |  |  | 5 – 10s | 761.64 | 309.12 |  |  |  |  |  |
| Fig. 3D | AUC | SD | -5 – 0s | 2.42 | 11.71 | 5 | two-way ANOVA | time x animal strain | F (1, 9) = 6.486 | 0.0314 |
|  |  |  | 5 – 10s | 202.88 | 101.33 |  |  | time | F (1, 9) = 19.91 | 0.0016 |
|  |  | FSL | -5 – 0s | 16.48 | 11.46 | 6 |  | animal strain | F (1, 9) = 6.057 | 0.0361 |
|  |  |  | 5 – 10s | 750.06 | 179.26 |  |  |  |  |  |
| Fig. 3F | AUC | SD | -5 – 0s | 20.77 | 16.64 | 5 | two-way ANOVA | time x animal strain | F (1, 9) = 5.496 | 0.0437 |
|  |  |  | 5 – 10s | 288.01 | 89.59 |  |  | time | F (1, 9) = 17.56 | 0.0023 |
|  |  | FSL | -5 – 0s | 10.84 | 17.96 | 6 |  | animal strain | F (1, 9) = 5.531 | 0.0432 |
|  |  |  | 5 – 10s | 956.69 | 245.99 |  |  |  |  |  |
| Fig. 4B | Number of calls (pre-DBS) | SD |  | 1.22 | 0.46 | 9 | t-test |  | t=1.119, df=12 | 0.2852 |
|  |  | FSL |  | 2.40 | 1.17 | 5 |  |  |  |  |
| Fig. 4C | Number of calls (DBS) | SD |  | 5.33 | 3.00 | 9 | Mann-Whitney test |  | U = 15.50 | 0.3701 |
|  |  | FSL |  | 11.40 | 7.51 | 5 |  |  |  |  |
| Fig. 4D | Number of calls (5s after DBS) | SD |  | 2.56 | 1.16 | 9 | Mann-Whitney test |  | U = 8 | 0.0465 |
|  |  | FSL |  | 30.20 | 15.07 | 5 |  |  |  |  |
| Fig. 4E | Number of calls (post-DBS) | SD |  | 2.56 | 1.67 | 9 | Mann-Whitney test |  | U = 3 | 0.0065 |
|  |  | FSL |  | 47.63 | 22.73 | 5 |  |  |  |  |
| Fig. 4F | Call length of pre-DBS | SD |  | 0.04 | 0.02 | 9 | t-test |  | t=1.088, df=12 | 0.2981 |
|  |  | FSL |  | 0.09 | 0.05 | 5 |  |  |  |  |
| Fig. 4G | Call length of DBS | SD |  | 0.16 | 0.09 | 9 | Mann-Whitney test |  | U = 15 | 0.3546 |
|  |  | FSL |  | 0.46 | 0.33 | 5 |  |  |  |  |
| Fig. 4H | Call length of 5s after DBS | SD |  | 0.09 | 0.04 | 9 | Mann-Whitney test |  | U = 7 | 0.0380 |
|  |  | FSL |  | 1.19 | 0.59 | 5 |  |  |  |  |
| Fig. 4I | Call length of post-DBS | SD |  | 0.13 | 0.11 | 9 | Mann-Whitney test |  | U = 3 | 0.0070 |
|  |  | FSL |  | 2.64 | 1.24 | 5 |  |  |  |  |
| Fig. 5B | Total A1 neurons/mm^2^ | SD |  | 127.48 | 7.55 | 10 | t-test |  | t=1.706, df=22 | 0.1021 |
|  |  | FSL |  | 145.87 | 7.33 | 14 |  |  |  |  |
| Fig. 5E | Total A2 neurons/mm^2^ | SD |  | 593.88 | 66.99 | 10 | Mann-Whitney test |  | U = 56.50 | 0.4450 |
|  |  | FSL |  | 515.39 | 46.35 | 14 |  |  |  |  |
| Fig. 5H | Total A6 neurons/mm^2^ | SD |  | 1017.91 | 43.62 | 10 | t-test |  | t=3.421, df=24 | 0.0022 |
|  |  | FSL |  | 798.64 | 32.37 | 14 |  |  |  |  |
| Fig. 5C | A1 cell group activation (%) | SD | stimulation | 60.71 | 7.89 | 7 | two-way ANOVA | animal strain x stimulation | F (1, 20) = 0.04763 | 0.8295 |
|  |  |  | sham | 17.00 | 4.58 | 3 |  | animal strain | F (1, 20) = 0.2368 | 0.6318 |
|  |  | FSL | stimulation | 65.09 | 2.33 | 11 |  | stimulation | F (1, 20) = 52.69 | <0.0001 |
|  |  |  | sham | 18.67 | 3.84 | 3 |  |  |  |  |
| Fig. 5F | A2 cell group activation (%) | SD | stimulation | 55.29 | 7.57 | 7 | two-way ANOVA | animal strain x stimulation | F (1, 19) = 2.613 | 0.1225 |
|  |  |  | sham | 50.00 | 17.50 | 3 |  | animal strain | F (1, 19) = 0.8696 | 0.3628 |
|  |  | FSL | stimulation | 61.10 | 2.59 | 10 |  | stimulation | F (1, 19) = 5.011 | 0.0374 |
|  |  |  | sham | 28.33 | 14.08 | 3 |  |  |  |  |
| Fig. 5I | A6 cell group activation (%) | SD | stimulation | 42.11 | 5.61 | 9 | two-way ANOVA | animal strain x stimulation | F (1, 21) = 2.319 | 0.1427 |
|  |  |  | sham | 36.33 | 4.18 | 3 |  | animal strain | F (1, 21) = 3.822 | 0.0640 |
|  |  | FSL | stimulation | 45.30 | 4.99 | 10 |  | stimulation | F (1, 21) = 0.5476 | 0.4675 |
|  |  |  | sham | 62.00 | 10.02 | 3 |  |  |  |  |
| Fig. 6B | PFC activated PV neurons/mm^2^ | SD | stimulation | 6.36 | 1.22 | 5 | two-way ANOVA | animal strain x stimulation | F (1, 13) = 16.18 | 0.0014 |
|  |  |  | sham | 6.01 | 1.12 | 3 |  | animal strain | F (1, 13) = 30.58 | <0.0001 |
|  |  | FSL | stimulation | 7.80 | 0.33 | 6 |  | stimulation | F (1, 13) = 13.39 | 0.0029 |
|  |  |  | sham | 15.12 | 0.94 | 3 |  |  |  |  |
| Fig. 6C | PFC cFOS+ neurons/mm^2^ | SD | stimulation | 102.23 | 15.37 | 6 | two-way ANOVA | animal strain x stimulation | F (1, 13) = 0.5687 | 0.4642 |
|  |  |  | sham | 64.28 | 16.40 | 3 |  | animal strain | F (1, 13) = 2.581 | 0.1322 |
|  |  | FSL | stimulation | 116.38 | 9.76 | 6 |  | stimulation | F (1, 13) = 2.347 | 0.1495 |
|  |  |  | sham | 103.47 | 17.33 | 3 |  |  |  |  |
| Fig. 6E | NAC activated PV neurons/mm^2^ | SD | stimulation | 3.34 | 0.52 | 5 | two-way ANOVA | animal strain x stimulation | F (1, 12) = 10.00 | 0.0082 |
|  |  |  | sham | 2.51 | 0.40 | 3 |  | animal strain | F (1, 12) = 10.12 | 0.0079 |
|  |  | FSL | stimulation | 3.35 | 0.74 | 5 |  | stimulation | F (1, 12) = 3.445 | 0.0882 |
|  |  |  | sham | 6.53 | 0.36 | 3 |  |  |  |  |
| Fig. 6F | NAC cFOS+ neurons/mm^2^ | SD | stimulation | 63.72 | 4.59 | 5 | two-way ANOVA | animal strain x stimulation | F (1, 12) = 12.18 | 0.0045 |
|  |  |  | sham | 67.99 | 12.17 | 3 |  | animal strain | F (1, 12) = 9.289 | 0.0101 |
|  |  | FSL | stimulation | 132.58 | 12.96 | 5 |  | stimulation | F (1, 12) = 9.518 | 0.0094 |
|  |  |  | sham | 63.33 | 7.31 | 3 |  |  |  |  |

| **Table S2 Post-hoc analysis (Bonferroni test)** | | | | | | | |
| --- | --- | --- | --- | --- | --- | --- | --- |
| **Figure** | **Subject** | **p** | | | | | |
| Fig. 3C | AUC |  |  | SD | | FSL | |
|  |  |  |  | -5 - 0 | 0 - 5 | -5 - 0 | 0 - 5 |
|  |  | SD | -5 - 0 |  |  |  |  |
|  |  |  | 0 - 5 | >0.999 |  |  |  |
|  |  | FSL | -5 - 0 | >0.999 |  |  |  |
|  |  |  | 0 - 5 |  | 0.012 | 0.006 |  |
| Fig. 3E | AUC |  |  | SD | | FSL | |
|  |  |  |  | -5 - 0 | 0 - 5 | -5 - 0 | 0 - 5 |
|  |  | SD | -5 - 0 |  |  |  |  |
|  |  |  | 0 - 5 | 0.695 |  |  |  |
|  |  | FSL | -5 - 0 | >0.999 |  |  |  |
|  |  |  | 0 - 5 |  | 0.005 | 0.002 |  |
| Fig. 3D | AUC |  |  | SD | | FSL | |
|  |  |  |  | -5 - 0 | 0 - 5 | -5 - 0 | 0 - 5 |
|  |  | SD | -5 - 0 |  |  |  |  |
|  |  |  | 0 - 5 | 0.454 |  |  |  |
|  |  | FSL | -5 - 0 | >0.999 |  |  |  |
|  |  |  | 0 - 5 |  | 0.005 | 0.001 |  |
| Fig. 3F | AUC |  |  | SD | | FSL | |
|  |  |  |  | -5 - 0 | 0 - 5 | -5 - 0 | 0 - 5 |
|  |  | SD | -5 - 0 |  |  |  |  |
|  |  |  | 0 - 5 | 0.486 |  |  |  |
|  |  | FSL | -5 - 0 | >0.999 |  |  |  |
|  |  |  | 0 - 5 |  | 0.008 | 0.002 |  |
| Fig. 6B | PFC activated PV neurons/mm^2^ |  |  | SD | | FSL | |
|  |  |  |  | Stimulation | Sham | Stimulation | Sham |
|  |  | SD | Stimulation |  |  |  |  |
|  |  |  | Sham | >0.999 |  |  |  |
|  |  | FSL | Stimulation | 0.456 |  |  |  |
|  |  |  | Sham |  | <0.0001 | 0.0002 |  |
| Fig. 6E | NAC activated PV neurons/mm^2^ |  |  | SD | | FSL | |
|  |  |  |  | Stimulation | Sham | Stimulation | Sham |
|  |  | SD | Stimulation |  |  |  |  |
|  |  |  | Sham | 0.747 |  |  |  |
|  |  | FSL | Stimulation | >0.999 |  |  |  |
|  |  |  | Sham |  | 0.003 | 0.008 |  |
| Fig. 6F | NAC cFOS+ neurons/mm^2^ |  |  | SD | | FSL | |
|  |  |  |  | Stimulation | Sham | Stimulation | Sham |
|  |  | SD | Stimulation |  |  |  |  |
|  |  |  | Sham | >0.999 |  |  |  |
|  |  | FSL | Stimulation | 0.0004 |  |  |  |
|  |  |  | Sham |  | >0.999 | 0.001 |  |
